# Bayesian parametric modeling of time to tuberculosis co-infection of HIV/AIDS patients at Jimma Medical Center, Ethiopia

**DOI:** 10.1038/s41598-022-20872-7

**Published:** 2022-10-01

**Authors:** Abdi Kenesa Umeta, Samuel Fikadu Yermosa, Abdisa G. Dufera

**Affiliations:** 1 Department of Statistics, Haramaya University, Dire Dawa, Ethiopia; 2 Department of Statistics, Jimma University, Jimma, Ethiopia

**Keywords:** Diseases, Risk factors, Mathematics and computing

## Abstract

Tuberculosis is the most common opportunistic infection among HIV/AIDS patients, including those following Antiretroviral Therapy treatment. The risk of tuberculosis infection is higher in people living with HIV/AIDS than in people who are free from HIV/AIDS. Many studies focused on prevalence and determinants of tuberculosis in HIV/AIDS patients without taking into account the censoring aspects of the time to event data. Therefore, this study was undertaken with aim to model time to tuberculosis co-infection of HIV/AIDS patients under follow-up at Jimma Medical Center, Ethiopia using Bayesian parametric survival models. A data of a retrospective cohort of 421 HIV/AIDS patients under follow-up from January 2016 to December 2020 until active tuberculosis was diagnosed or until the end of the study was collected from Jimma Medical Center, Ethiopia. The analysis of the data was performed using R-INLA software package. In order to identify the risk factors which have association with tuberculosis co-infection survival time, Bayesian parametric accelerated failure time survival models were fitted to the data using Integrated Nested Laplace Approximation methodology. About 26.37% of the study subjects had been co-infected with tuberculosis during the study period. Among the parametric accelerated failure time models, the Bayesian log-logistic accelerated failure time model was found to be the best fitting model for the data. Patients who lived in urban areas had shorter tuberculosis co-infection free survival time compared to those who lived in rural areas with an acceleration factor of 0.2842. Patients who smoke and drink alcohol had also shorter tuberculosis co-infection survival time than those who do not smoke and drink alcohol respectively. Patients with advanced WHO clinical stages(Stage III and IV), bedridden functional status, low body mass index and severe anemic status had shorter tuberculosis co-infection survival time. Place of residence, smoking, drinking alcohol, larger family size, advanced clinical stages(Stage III and Stage IV), bedridden functional status, CD4 count ($\le$ 200 cells/mm^3^ and 200–349 cells/mm^3^), low body mass index and low hemoglobin are the factors that lead to shorter tuberculosis co-infection survival time in HIV/AIDS patients. The findings of the study suggested us to forward the recommendations to modify patients’ life style, early screening and treatment of opportunistic diseases like anemia , as well as effective treatment and management of tuberculosis and HIV co-infection are important to prevent tuberculosis and HIV co-infection.

## Introduction

Tuberculosis is one of the infectious diseases that affects the lungs and other sites^1^. Tuberculosis has been the main public health problem affecting millions worldwide and it remains the top infectious killer in the world causing close to 4000 lives a day^2^. Around 10.0 million peoples estimated to have developed TB disease in 2019 worldwide, and there were around 1.2 million TB deaths among HIV-negative people and an additional 208, 000 deaths among people living with HIV^3^.

Tuberculosis is the most common opportunistic infection among HIV positive people including those under ART treatment follow-up, and it is the major cause of HIV -related death^4^. UNAIDS report of 2018 showed that Sub-Saharan Africa is the hardest hit region of the world, as it has around 70% of all people living with HIV/AIDS and TB co-infection in the world^5^. Global Tuberculosis report of 2020 showed that Ethiopia is one the countries with the highest TB/HIV co-infection prevalence^3^.

The HIV virus infects CD4 cells causing reduction of the number of immune cells which causes the body fail to control viral multiplication which increases the chance of opportunistic infection with tuberculosis being the most common opportunistic infection at HIV diagnosis^6^. The treatment outcome of HIV-positive following ART treatment has remarkably changed with a a reduction of plasma viral copies and an increase of CD4 counts^7^. It had been reported that the ART treatment has reduced the incidence of TB in HIV patients by about 70–90%^8^. Even with the advantages of ART treatment, still HIV/AIDS patients following ART treatment develop TB with about prevalence rate of 2.5–30.1^6^.

Though, Tuberculosis can affect everyone, the risk of Tuberculosis infection is higher in people living with HIV than in people who are free from HIV^9^. Studies revealed that certain HIV-infected people develop TB, while others do not. This phenomenon shows that being HIV positive is not the only factor for being infected with TB^10^. There are various factors that increase the chance of TB infection among HIV/AIDS patients including CD4 cell count and the number of viral loads^11,12^, household family size, cigarette smoking, baseline CD4 cell counts, WHO clinical stages, having a history of diabetics^13^, and etc. However, these factors have not been studied in the context of survival analysis, where association between risk factors and time to TB co-infection might be of interest.

Majority of the study focused on prevalence and predictors of TB in HIV patients. In order to determine the important determinants of TB co-infection in HIV patients, most of the methodologies in the literature used logistic regression with outcome being the TB’s viability through follow up time of HIV/AIDS patients taking ART treatment^10,14,15^. In logistic regression, our interest is to study how risk factors were associated with the presence or absence of a disease( or an event) without taking into account the effect of time^16^. These approaches help to provide odds ratios for significant variables associated with the risk of TB infection but rejects the censoring aspects of time-to-event data. Researchers have also used non-parametric survival methods and Cox regression models to identify risk factors associated with TB co-infection in HIV/AIDS patients. Non parametric survival methods have two disadvantages. Its first disadvantage is that we cannot incorporate covariates, meaning that it is difficult to describe how individuals differ in their survival functions. The other disadvantage of non-parametric method is that the survival functions are not smooth^17,18^. A parametric survival model is a model where the survival time is assumed to follow a particular distribution such as exponential (a special case of the Weibull), Weibull, log-logistic, log-normal and gamma. Therefore, the aim of this study was to model the predictors of time to TB co-infection in HIV/AIDS patients following ART treatment using Bayesian parametric survival analysis approach based on INLA methodology. There have been advances in computational and modeling techniques using Bayesian approach of survival data^19^. Due to the complex likelihood functions to accommodate censoring, survival models are generally very difficult to fit. Bayesian approach to survival analysis may overcome this by using the MCMC techniques and other numerical integration methods like INLA^20^.

The integrated nested Laplace approximation method for approximate Bayesian inference was developed by Rue, Martino, and Chopin as an alternative to the MCMC method^21^. INLA is an alternative method for Bayesian inference on latent Gaussian models when the focus is on posterior marginal distributions. It substitutes MCMC methods with accurate, deterministic approximations to posterior marginal distributions. Integrated nested Laplace approximation provides a fast and exact approach to fitting latent Gaussian models which include many statistical models, including survival models^22^.

Survival models can be written into a latent Gaussian model which allows us to perform Bayesian inference using integrated nested Laplace approximations^19^. Survival analysis consists of a great body of work using latent Gaussian models and it is one of the statistical models on which INLA has been successfully applied^23,24^. The main advantage of INLA over MCMC techniques is its simplicity of computation^24^. Using INLA results are generated within seconds and minutes even for models with a large dimensional latent field, where as MCMC algorithm would take hours or even days. The other advantage of INLA is that INLA treats latent Gaussian models in a unified way, thus allowing greater automation of the inference process. Even though Bayesian approaches to the analysis of survival data can provide a number of benefits, they are less widely used than the classical approaches^25^.

Even though Bayesian approaches to the analysis of survival data can provide a number of benefits, they are less widely used than the classical approaches^25^. Therefore, the motivation to apply Bayesian Survival Analysis for this study stems from the above mentioned advantages of Bayesian survival analysis approach over the classical survival analysis approach.

## Methods

### Study area and period

This study was conducted at Jimma Medical Center, South-west of Ethiopia. Jimma Medical Center is one of the oldest hospitals in Ethiopia and it is the only teaching referral hospital in South-west Ethiopia with 800 bed capacity and serving the majority of peoples living in Jimma city and its surroundings. The total number of population of the study was 3069. The study was conducted from January 2016 to December 2020.

### Data description

The nature of the data set used for this study was survival data. In the data set, time until active TB infection was clinically diagnosed in HIV/AIDS patients was investigated. This study investigated the time at which patients were diagnosed and tested positive with TB.

### Inclusion and exclusion criteria

All adult HIV/AIDS patients following ART treatment and who were 18 years old and above during the study period and TB free at the inception of the study with at least two follow up period were included in the study. Patients whose date of TB co-infection was unknown were excluded from the study. Also, patients with insufficient information about one of the variables in the study were not included.

### Study design, population and sample size

A data of a retrospective cohort of adult HIV/AIDS patients from January 2016 to December 2020 until active TB was clinically diagnosed or until the end of the study was collected from ART clinic of Jimma Medical Center, Ethiopia.This study investigated the time at which patients were diagnosed and tested positive with TB). In this study, the source population was all adult HIV/AIDS patients who were 18 years old and above at Jimma Medical Center. There were a total number of 3069 HIV/AIDS pateints. Among the total patients 421 of the patients were included in the study based on the inclusion and exclusion criteria.

### Study variables

#### Dependent variable

The dependent variable for this study was time to active TB infection in HIV/AIDS patients at Jimma Medical Center. Time is measured in months and it is the difference between time of ART initiation and TB infection.

*Starting time:* Starting Timeis the time at which the patient initiates ART treatment.

*End time:* is the time at which the event occurred, when the HIV patients patients get infected with TB or was lost to follow-up before the completion of the study,or completed the study duration without any events(censored observations).

#### Independent variables

The independent or the predictor variables which were assumed to have effect on time to TB infection in HIV/AIDS patients were age, sex, place of residence, family size, alcohol usage status, smoking status, marital status, education level, WHO clinical stages, functional status, CD4 count, body mass index and hemoglobin level.

### Methods of data analysis

#### Survival data analysis

Survival analysis is a collection of statistical techniques for data analysis for which the outcome variable of interest is the time until an event occurs. By time, we mean years, months, weeks, or days from the beginning of follow-up of an individual until an event occurs. By event, we mean death, disease incidence, relapse from remission, recovery from disease or any designated experience of interest that may happen to an individual. Censoring is one of the common features that makes survival analysis unique from another statistical analysis. Censoring is present when we have some information about a subject’s event time, but we don’t know the exact event time^26^. The general reasons why censoring might occur are: a subject does not experience the event before the study ends, the patient is lost to follow-up during the study period, or the patient withdraws from the study.

### Survival function

Assume that the survival time, T, is a continuous random variable. The distribution of T can be described by the usual cumulative distribution function

$$\begin{aligned} F(t) = P(T \le t) = \int _{0}^{t}f(u)du, \text {where}; t \ge 0 \end{aligned}$$

which is the probability that a subject from the population will die (or a specific event of interest for a subject has occurred) before time t^27^. The corresponding density function of T is

$$\begin{aligned} f(t) = \frac{\partial }{\partial t}F(t) \end{aligned}$$

In survival analysis, it is common to use the survival function

$$\begin{aligned} S(t) = P(T \ge t) = 1 - F(T), t \ge 0 \end{aligned}$$

The relationship between f(t) and S(t) is given as follows;

$$\begin{aligned} f(t) = \frac{\partial }{\partial t}F(t) = \frac{\partial }{\partial t}\left( 1 - S(t)\right) = \frac{- \partial }{\partial t}S(t) \end{aligned}$$

### Hazard function

It is also of interest, in analyzing survival data, to assess which periods having high or low chances of the event among those still active at the certain time. A suitable method to characterize such risks is the hazard function., h(t), defined by the following equation^27^.

$$\begin{aligned} h(t) = \lim \limits _{S \rightarrow 0}\frac{P(t\le T \le t+s/T\ge t)}{S} \end{aligned}$$

It is the instantaneous rate of failure (experiencing the event) at the time t given that a subject is alive at the time t. The definition of the hazard function implies that

$$\begin{aligned} h(t) = \frac{f(t)}{S(t)} = -\frac{\partial }{\partial t}log\left( S(t)\right) \end{aligned}$$

A related quantity is the cumulative hazard function, H(t), defined by

$$\begin{aligned} H(t) = \int _{0}^{t}h(u)du = -log\left( S(t)\right) \end{aligned}$$

And thus,

$$\begin{aligned} S(t) = exp\left( -H(t)\right) = exp\left( -\int _{0}^{t}h(u)du\right) \end{aligned}$$

### The Kaplan-Meier estimator of survival function

Kaplan-Meier method also known as product limit estimator is widely used tool in survival analysis in dealing with censored data. It is a method for time to event data at each time point when a particular event takes place. By using this method, we can make comparisons of the survival or failure rates between two or more groups in order to see either the effect of particular treatments on the survival time of the patients or to show the survivor function risk groups^28^. To estimate the survivor function, S(t), without covariates, we can use the Kaplan Meier estimator. This method does not rely on distributional assumptions (distribution free method) and hence categoorized as non-parametric technique.

Let there be n individuals with observed survival times $$t_{1}, ...,t_{n}$$ and r be death times amongst the individuals, where $$r \ge n$$, j = 1,…,r. The r ordered death times are $$t_{(1)}< t_{(2)}< ... < t_{(r)}$$. Let $$n_{j}$$ denotes the number of individual who are alive just before time $$t_{(j)}$$ , including those who are about to die at this time, and let $$d_{j}$$ denotes the number who die at this time. The Kaplan-Meier estimator of the survival function at any time in the $$k^{th} $$ time interval from $$t_{(k)}$$ to $$t_{(k+1)}$$, k = 1,…, r is given by^29^.

$$\begin{aligned} {\hat{S}}_{(t)} = \prod _{j = 1}^{k}\left( \frac{n_{j} - d_{j}}{n_{j}}\right) \end{aligned}$$

### Parametric survival models

In a parametric survival models, survival time is assumed to follow a known distribution^30^. Parametric models play an important role in Bayesian survival analysis, since many Bayesian analyses in practice are carried out using parametric models and parametric modeling offers straightforward modeling and analysis techniques^20^.

### Ethics approval and consent to participate

Letter of ethical clearance was obtained from Department of Statistics of Jimma University and submitted to Jimma Medical Center to get permission to undertake the research. This study was developed in accordance with established legislation and complies with the norms of good clinical practice, and informed consent was being not necessary as personal identifying information was kept separate from the research data.

## Parametric proportional hazard models

Let h(t/x)) be the hazard function at time t for a subject given the covariate vector x = ($x_{1},\ldots , x_{p})^{T}$. The basic model proposed by Cox is as follows:

$$\begin{aligned} h(t/x) = h_{0}(t)exp(\beta _{1}x_{1} +\cdots + \beta _{p}x_{p}) \end{aligned}$$

where $h_{0}(t)$ is the baseline hazard function and $\beta _{i}$’s are the unknown regression parameters to be estimated. In parametric proportional hazard model, the baseline hazard function $h_{0(t)}$ is assumed to follow a specific distribution when a fully parametric PH model is fitted to the data. The hazard ratio is hence given by HR = $exp\left( \beta _{1}X_{1} + \beta _{2}X_{2} + \cdots + \beta _{p}X_{p}\right)$.

### Accelarated failre time models

Although parametric PH models are very useful to analyze survival data, there are relatively few probability distributions for the survival time that can be used with these models^31^. In these situations, the accelerated failure time model (AFT) is an alternative to the PH model for the analysis of survival time data. Under AFT models we measure the direct effect of the explanatory variables on the survival time instead of hazard, as we do in the PH model. This characteristic allows for an easier interpretation of the results because the parameters measure the effect of the covariates on the survival time.

In accelerated failure time (AFT) models, the natural logarithm of the survival time, log(t), is expressed as a linear function of the covariates, which yields therefore a linear model:

$$\begin{aligned} log(t_{i}) = \mu + \beta _{1}x_{1i} + \beta _{2}x_{2i} + \cdots + \beta _{p}x_{pi} + \sigma  \varepsilon   _{i} = X_{i}\beta + z_{i} \end{aligned}$$

We interpret the effect of the AFT model as the change in the time scale by a factor of exp(**xj**$\beta$). Based on whether this factor is greater or less than 1, survival time is interpreted to either accelerate or decelerate. Accelerated failure time does not imply a positive acceleration of time with the increase of a covariates but rather a deceleration, or, in other words, an increase in the expected waiting time until failure. AFT models have the opposite sign from similar estimates in proportional hazard models, due to the fact that the PH models predict the hazard and the AFT model predicts time.

An advantage of the AFT approach is that the effect of the covariates is described in absolute terms (i.e. number of months or years) instead of in relative terms (i.e. a hazard ratio). The acceleration factor is the central measure of association obtained in AFT models and allows you to evaluate the effect of covariates on the survival time.

### Bayesian modeling approach for survival data

The Bayesian paradigm is based on specifying a probability model for the observed data D, given a vector of unknown parameters $\theta$, leading to the likelihood function L($\theta$/D). Then we assume that $\theta$ is random and has a prior distribution denoted by $\pi (\theta )$. Inference concerning $\theta$ is then based on the posterior distribution^20^ , which is obtained by Bayes’ theorem. The posterior distribution of $\theta$ is given by

$$\begin{aligned} \pi (\theta /D) = \frac{L(\theta /D)\pi (\theta )}{\int _{\varTheta }L(\theta /D)\pi (\theta )d \theta } \end{aligned}$$

where $\varTheta$ denotes the parameter space of $\theta$.

The quantity $m(D) = \int _{\varTheta }L(\theta /D)\pi (\theta )d \theta$ is the normalizing constant of $\pi (\theta /D)$ , and is often called the marginal distribution of the data or the prior predictive distribution. In most models and applications, *m*(*D*) does not have an analytic closed form, and therefore $\pi (\theta /D)$ does not have a closed form. The Bayesian survival analysis approach considers the parameters of the model as random variables and requires that prior distributions specified for them and data are considered as fixed^32^.

### Likelihood function in Bayesian survival analysis

Suppose we observe n independent vectors of ($T_{i}$, $\delta _{i}$), where $T_{i}$ is time to the event and $\delta _{i}$ is indicator variable telling us whether $T_{i}$ is censored or not, i.e, $T_{i} = 0$ for censored observation($\delta _{i} = 0$) and $T_{i} = 1$ for uncensored observation($\delta _{i} = 1$).

The likelihood function of the set of unknown parameters $\theta$ in the presence of right censoring is given as

$$\begin{aligned} L(\theta /D) = \prod _{i=1}^{n}f(t_{i},\theta )^{I(\delta _{i} = 0)} * S(t_{i},\theta )^{I(\delta _{i} = 1)} *\pi (\theta /D) \end{aligned}$$

### The integrated nested laplace approximation methodology for Bayesian inference

For long time, Bayesian statistical inference has relied on MCMC methods to compute the joint posterior distribution of the model parameters which is usually computationally very expensive^33^. An alternative approach and fast estimation methods to MCMC which allows user to easily perform approximate Bayesian inference using Integrated Nested Laplace Approximation was proposed by Havard Rue, Martino, and Chopin^19^. INLA computes posterior marginals for each component in model, from which posterior expectation and standard deviations can easily be found.

### The integrated nested laplace approximation procedure

In order to approximate the posterior marginals of $\pi (xi/y), \pi (\theta /y)$ and $\pi (\theta _{j}/y)$ the latent Gaussian models is used^19^. Latent Gaussian models are subset of all Bayesian additive models with a structured additive predictor say $\eta _{i}$. In these models, the observed variable $y_{i}$ is assumed to belong to an exponential family, where the mean $\mu _{i}$ is linked to this structured additive predictor $\eta _{i}$ through a link function g(.), so that $g(\mu _{i}) = \eta _{i}$. The structured additive predictor $\eta _{i}$ accounts for effects of various covariates in an additive way:

$$\begin{aligned} \eta _{i} = \alpha + \sum _{j=1}^{n_{f}}f^{(j)}(u_{ji}) + \sum _{k =1}^{n_{\beta }}\beta _{k}Z_{ki} + \varepsilon \end{aligned}$$

Here, the $\left\{ f^{j}(.)\right\}$ are unknown functions of covariates **u**, the $\left\{ \beta _{k}\right\}$ represent the linear effect of covariates **z** and the $\varepsilon _{i}$’s are unstructured terms. A Gaussion prior is assigned to $\alpha , \left\{ f^{j}(.)\right\} , \left\{ \beta _{k}\right\}$ and $\varepsilon _{i}$. We denote $\pi (./.)$ as the conditional density of its arguments, and let **x** denote the vector of all n Gaussian variables $\eta _{i}, \alpha , \left\{ f^{j}(.)\right\}$ and $\left\{ \beta _{k}\right\}$, and $\theta$ denotes the vector of hyper-parameters, which are not necessarily Gaussian. The density $\pi (x/\theta _{1})$ is Gaussian with(assumed) zero mean and precision matrix $Q(\theta _{1})$ with hyperparameter $\theta _{1}$.

The distribution for the $n_{d}$ observational variables **y** = $\left\{ y_{i}:i \varepsilon I\right\}$ is denoted by $\pi (y/x, \theta _{2})$ and we assume that $\left\{ y_{i}:i \varepsilon I\right\}$ are conditionally independent given x and $\theta _{2}$. For simplicity, we denote by $\theta$ = $\left( \theta _{1}^{T}, \theta _{2}^{T}\right) ^{T}$ with dim($\theta$) = m. The posterior then reads( for non singular **Q**($\theta$)),

$$\begin{aligned} \pi \left( x, \theta /y\right) = \pi (\theta )\pi (x/\theta )\prod _{ i \varepsilon I}\pi (yi/xi, \theta ) \end{aligned}$$

The imposed linear constraints(if any) are denoted by **Ax** = **e** for a kxk matrix A of rank k. The main aim is to approximate the posterior marginals of the latent field, $\pi (xi/y)$ and the posterior marginals of the hyperparameters , $\pi (\theta /y)$ and $\pi (\theta _{j}/y)$. We can write the posterior marginal of interest as

$$\pi (x_{i}) = \int \pi (x_{i}/\theta ,y)\pi (\theta /y)d\theta $$

$$\pi (\theta _{j}) = \int \pi (\theta /y)d\theta _{-j}$$

The importance of INLA is to use the above form to construct nested approximations, as this approach makes Laplace approximations very accurate when applied to latent Gaussian models.

$${\tilde{\pi }}(x_{i}/y) = \int \pi (x_{i}/\theta ,y){\tilde{\pi }}(\theta /y)d\theta )$$

$${\tilde{\pi }}(\theta _{j}/y) = \int {\tilde{\pi }}(\theta /y)d\theta _{-j}$$

Here, ${\tilde{\pi }}(./.)$ is an approximated( conditional) density of its arguments. Approximations to $\pi (x_{i}/y)$ are computed by $\pi (\theta /y)$ and $\pi (x_{i}/\theta , y)$ and using numerical integration to integrate out $\theta$. The approximation of $\pi (\theta _{j}/y)$ is computed by integrating out $\theta _{-j}$ from ${\tilde{\pi }}(\theta /y)$. The posterior marginal $\pi (\theta )$ of the hyperparameters $\theta$ is approximated using a Laplace approximation

$$ {\tilde{\pi }}(\theta /y) \propto \frac{\pi (x, \theta , y)}{\tilde{\pi _{G}}(x/\theta ,y)}\mid x = x^{*}(\theta )$$

### Prior distributions in INLA

Bayesian statistical inference depends on the posterior distribution which is obtained by updating the prior beliefs by new evidence. Prior distribution can be broadly classified into non-informative, weakly informative and informative prior distributions. Non-informative prior distributions, also known as objective prior distributions, are designed to have minimal impact on the posterior distribution so that the data alone can be the source of inference^34^. The non-informative prior distribution often produce the same results as maximum likelihood estimates. On the other hand, the informative prior distributions that aim to construct a prior distribution that reflect the current knowledge on the values of the parameters and the uncertainties that surround the knowledge about the parameters in question^35^. In INLA, it is assumed that fixed effects follow Gaussian distribution with mean zero and small number of precision matrix **Q**($\theta _{1}$) and only the parameters in the precision matrix of the random effect need a prior which was considered as a hyper-parameter^36^. For this study, Gaussian prior distribution (non-informative) with mean zero and variance equal to 1000(precision equal to 0.001) was used for the fixed effects and the intercept^21^. And for hyper-parameters a non-informative prior of Gamma distribution prior is a common non -informative prior to be assigned^22^. In INLA, the Latent component of the model, $\eta _{i} = \beta _{0}+\beta _{1}z_{1}+ \cdots +\beta _{p}z_{p}$ must follow a Gaussion distribution^22^. In this study, it was assumed that fixed effect(coefficients) associated with covariates have a Normal distribution with mean 0 and variance $10^{2}$, i.e, $\beta _{p}$, p = 0,…, i.e, $\beta _{p} \sim N(0, 10^{2})$^20^. Then for this study, to complete the model we have assigned a non-informative Gamma prior for for the hyperparameter of the model $\tau _{i} \sim \Gamma (a, b)$ and $\alpha \sim \Gamma (a, b)$ with a =1 and b = 0.001 which is similar with prior distribution used by many researchers worked on Bayesian survival analysis^19–21^.

### Bayesian parametric survival models

#### Exponential model

The exponential model is the most fundamental parametric model in survival analysis^20^. Suppose we have independent identically distributed (i.i.d.) survival times **t** = ($t_{1}, t_{2},\ldots , t_{n}$)’, each having an exponential distribution with parameter $\lambda$. Denote the censoring indicators by $\delta$ = ($\delta _{1}, \delta _{2}, \ldots , \delta _{n}$)’, where $\delta _{i} = 0$ if $T_{i}$ is right censored and $\delta _{i} = 1$ if $T_{i}$ is a failure time. Let $f(t_{i}/ \lambda ) = \lambda exp\left( - \lambda t_{i}\right)$ denote the density for $t_{i}$, $S(t_{i}/ \lambda ) = exp \left( - \lambda t_{i}\right)$ denotes the survival function. We build a regression model by introducing covariates through $\lambda$, and write $\lambda _{i} = \varphi (x_{i}'\beta )$, where $x_{i}$’is a p x 1 vector of covariates, $\beta$ is a p x 1 vector of regression coefficients, $\varphi (.)$ and is a known functionand D = (n,**t,X,** $\delta$) denotes the observed data for regression model Using these, we get the likelihood function^19^.

$$\begin{aligned} L(\beta /D)= & {} \prod _{i =1}^{n}f(t_{i}/\lambda _{i})^{\delta _{i}}S(t_{i}/\lambda _{i})^{1 - \delta _{i}}\\= & {} exp\left\{ \sum _{i =1}^{n}\delta _{i}x_{i}'\beta \right\} exp\left\{ -\sum _{i =1}^{n}t_{i}\text {exp}(x_{i}'\beta )\right\} \end{aligned}$$

Suppose we specify a normal prior for $\beta$ with mean $\mu _{0}$ and variance $\sigma _{0}^{2}$. Then the posterior distribution of $\beta$ is given by

$$\begin{aligned} \pi (\beta /D) \varpropto L(\beta /D)\pi (\beta /\mu _{0}, \sigma _{0}) \end{aligned}$$

where $\pi (\beta /\mu _{0}, \sigma _{0})$ is the normal density with mean $\mu _{0}$ and variance $\sigma _{0}^{2}$.

#### Weibull model

The Weibull model is perhaps the most widely used parametric survival model^20^. Suppose we have independent identically distributed survival times **t** = ($t_{1}, t_{2}, ..., t_{n}$)’, each having a Weibull distribution, denoted by $\omega (\alpha , \gamma )$. It is often more convenient to write the model in terms of the parameterization $\lambda = log(\gamma )$, leading to$f(t_{i}/\alpha , \lambda ) = \alpha t_{i}^{\alpha - 1}exp(\lambda - exp(\lambda )t_{i}^{\alpha })$ Let $S(t_{i}/\alpha , \lambda ) = exp\left( -exp(\lambda ) t_{i}^{\alpha }\right)$ denote the survival function. We can write the likelihood function of ($\alpha , \lambda$) as

$$\begin{aligned} L(\alpha , \lambda /D)= & {} \prod _{i =1}^{n}f(t_{i}/\alpha ,\lambda )^{\delta _{i}}S(t_{i}/\alpha ,\lambda )^{(1 - \delta _{i})}\\= & {} \alpha ^{d} exp\left\{ \begin{aligned}d \lambda + \sum _{i = 1}^{n}(\delta _{i}(\alpha - 1))log(t_{i}) - exp(\lambda )t_{i}^{\alpha }\end{aligned}\right\} \end{aligned}$$

Where d = $\sum _{i = 1}^{n}\delta _{i}$ and $\delta$ is the indicator variable taking value 1 if ti is failure time and 0 if $t_{i}$ is right censored.

To build the Weibull regression model, we introduce covariates through $\lambda$ and write $\lambda _{i} = x_{i}'\beta$. Where $x_{i}$ is a px1 vector of covariates, $\beta$ is a px1 vector of regression coefficients. Assuming a normal prior with parameters $\left( \mu _{0},\sigma _{0}^{2}\right)$ for $\lambda$ and gamma prior with parameters $\left( \alpha _{0}, \kappa _{0}\right)$, the joint posterior distribution of $(\alpha , \lambda )$ is given by

$$\begin{aligned} \pi (\beta , \alpha /D) \varpropto \alpha ^{\alpha _{0} + d - 1}exp\left\{ \begin{aligned}\sum _{i =1}^{n}\left( \delta _{i}x_{i}'\beta + \delta _{i}(\alpha - 1)log(t_{i})\right) -\left( t_{i}^{\alpha }exp(x_{i}'\beta )\right) - \kappa _{0}\alpha - \frac{1}{2}(\beta - \mu _{0})\frac{1}{\sigma _{0}^{2}}(\beta - \mu _{0})\end{aligned}\right\} \end{aligned}$$

Where D = $\left( n, t, {\textbf {x}},\delta \right)$ denote the observed data for regression model.

#### Log-logistic model

The log-logistic model possesses a rather supple functional form^37^. The Log-logistic distribution is among the parametric survival models where the hazard rate initially increases and then decreases. If we have independent identically distributed survival times **t** =($t_{1}, t_{2}, ... , t_{n}$)’, each having an log-logistic distribution, denoted by T $\sim LL(\alpha , \lambda )$, with density

$$\begin{aligned} f(t_{i}/\alpha , \lambda ) = \frac{\alpha \lambda ^{\alpha }t_{i}^{\alpha - 1}}{(t_{i}^{\alpha }+\lambda ^{\alpha })^{2}} \end{aligned}$$

for $$\alpha> 0, \lambda > 0$$ and $$t \ge 0$$.

And, the survival function is given by

$$\begin{aligned} S(t_{i}/\alpha , \lambda ) = \frac{\lambda ^{\alpha }}{(t_{i}^{\alpha } + \lambda ^{\alpha })} \end{aligned}$$

for $$t > 0$$.

We can write the likelihood function of ($\alpha , \lambda$) as

$$\begin{aligned} L(\alpha , \lambda |D)= & {} \prod _{i = 1}^{n}f(t_{i}/\alpha , \lambda )^{\delta _{i}}S(t_{i}/\alpha , \lambda )^{(1 - \delta _{i})}\\= & {} \alpha ^{d}\lambda ^{n \alpha }t_{i}^{(\alpha - 1)d}(t_{i}^{\alpha } + \lambda ^{\alpha })^{-d} \end{aligned}$$

Where d = $\sum _{i =1}^{n}\delta _{i}$ and Where $\delta$ is the indicator variable taking value 1 if ti is failure time and 0 if ti is right censored.

To build the regression model, we introduce covariates through $\lambda$, and write $\lambda _{i} = x_{i}'\beta$. Where $x_{i}'$ is px1 vector of covariates, and $\beta$ is px1 regression coefficients. If we assume gamma prior with parameters ($\alpha _{0}, \kappa _{0}$) for $\alpha$, we will have the following joint posterior

$$\begin{aligned} \pi (\beta , \alpha /D) \varpropto \alpha ^{d}(n \alpha +\alpha _{0} - 1)\left\{ \begin{aligned}exp(x_{i}\beta )+ d(\alpha - 1)exp(t_{i}) - dexp(t_{i}^{\alpha } + \lambda ^{\alpha }) + log(\kappa _{0}x_{i}\beta ) \end{aligned}\right\} \end{aligned}$$

## Log-normal model

Another commonly used parametric survival model is the log-normal model^20^. For this model, we assume that the logarithms of the survival times are normally distributed. If $t_{i}$ has a log-normal distribution with parameters ($\mu , \sigma$) , denoted by $\iota N(\mu ,\sigma )$, then

$$\begin{aligned} f(t_{i}/\mu , \sigma ) = (2\pi )^{-1/2}(t_{i}\sigma )^{-1}\text {exp}\left\{ \frac{-1}{2\sigma ^2} (log(t_{i}) - \mu )^{2}\right\} \end{aligned}$$

The survival function is given by

$$\begin{aligned} S(t_{i}/\mu , \sigma ) = 1 - \varPhi \left( \frac{log(t_{i}) - \mu }{\sigma }\right) \end{aligned}$$

We can thus write the likelihood function of ($\mu , \sigma$) as

$$\begin{aligned} L(\mu ,\sigma /D) = \prod _{i =1}^{n}f(t_{i}|\mu ,\sigma )^{\delta _{i}}S(t_{i}|\mu ,\sigma )^{1 - \delta _{i}} \end{aligned}$$

Then,

$$\begin{aligned} L(\mu ,\sigma /D) = (2\pi )^{-1/2}\text {exp}\left\{ - \frac{1}{2\sigma ^{2}}\sum _{i =1}^{n}\delta _{i}(log(t_{i}) - \mu )^{2}\right\} X \prod _{i =1}^{n}t_{i}^{-\delta _{i}}\left( 1-\varPhi \left( \frac{log(t_{i}) - \mu }{\sigma }\right) \right) ^{1 - \delta _{i}} \end{aligned}$$

To construct the regression model, we introduce covariates through $\mu$, and write $\mu _{i} = x_{i}'\beta$. Assuming $\beta /\tau \sim N_{p}(\mu _{0}, \tau ^{-1} \varsigma _{0})$, the joint posterior for $\beta , \tau$ is given by

$$\begin{aligned}&\pi (\beta , \tau |D) \varpropto \tau ^{\frac{\alpha _{0} + d }{2} - 1 exp\left\{ -\frac{\tau }{2} \left[ \begin{aligned}\sum _{i =1}^{n}\delta _{i}\left( log(t_{i}) - x_{i}')\beta \right) ^{2} + (\beta - \mu _{0})'\frac{1}{\sigma _{0}^{2}}(\beta - \mu _{0}) + \lambda _{0}\end{aligned}\right] \right\} }\\&\quad X \prod _{i =1}^{n}t_{i}^{-\delta _{i}}\left( 1- \varPhi \left( \tau ^{1/2}(log(y_{i} - x_{i}'\beta ))\right) \right) ^{1 - \delta _{i}} \end{aligned}$$

### Gamma model

The gamma model is a generalization of the exponential model^20^. For this model, $t_{i} \sim \zeta (\alpha , \lambda )$ and its density function is given by:

$$\begin{aligned} f(t_{i}/\alpha ,\lambda ) = \frac{1}{\Gamma {(\alpha )}}t_{i}^{\alpha - 1}exp(\alpha \lambda - t_{i}exp(\lambda )) \end{aligned}$$

The survival function is given by

$$\begin{aligned} S(t_{i}/\alpha , \lambda ) = 1 - \frac{1}{\Gamma (\alpha )}\int _{0}^{t_{i}\text {exp}(\lambda )}u^{\alpha - 1}\text {exp}(-u)du, \end{aligned}$$

We can thus write the likelihood function of ($\alpha , \lambda$) as

$$\begin{aligned} L(\alpha , \lambda |D) = \prod _{i =1}^{n}f(t_{i}|\alpha , \lambda )^{\delta _{i}}S(t_{i}|\alpha , \lambda )^{(1 - \delta _{i})} \end{aligned}$$

To construct the regression model, we introduce covariates through $\lambda$, and write $\lambda _{i} = x_{i}'\beta$. Assuming $\beta \sim N(\mu _{0}, \sigma _{0}^{2})$, we are lead to the joint posterior

$$\begin{aligned} \pi (\beta , \alpha /D) \varpropto \frac{\alpha ^{\alpha _{0} -1}}{(\Gamma (\alpha ))^{d}}exp\left\{ \begin{aligned}\sum _{i =1}^{n}\delta _{i}\left[ \begin{aligned}\alpha (x_{i}'\beta ) + log(t_{i})\\ - t_{i}exp(x_{i}'\beta )\end{aligned}\right] \end{aligned}\right\} X \prod _{i =1}^{n}t_{i}^{-\delta _{i}}(1 - IG(\alpha , t_{i}(x_{i}'\beta )))^{1 - \delta _{i}}\\ X \text {exp}\left( -\kappa _{0} \alpha - \frac{1}{2\sigma _{0}^{2}}(\beta -\mu _{0})'(\beta - \mu _{0})\right) \end{aligned}$$

Where, IG = $\frac{1}{\Gamma (\alpha )}\int _{0}^{t_{i}exp(\lambda )}u^{\alpha - 1}exp(-u)du$ is the incomplete gamma function.

### Model comparison methods

Integrated Nested Laplace Approximation computes a number of Bayesian criteria for model assessment and model selection^38^. Model selection criteria will be of help when selecting among different models. The following methods of model selection techniques was used in this study.

## Marginal likelihood

The marginal likelihood of a model is the probability of the observed data under a given model^39^. The marginal likelihood approximation provided by INLA is computed as.

$$\begin{aligned} {\tilde{\pi }}(y) = \int \frac{\pi (\theta ,x,y)}{{\tilde{\pi }}_{G}(x/\theta ,y)}|_{x = x^{*}(\theta )}d\theta \end{aligned}$$

### Information-based criteria (DIC and WAIC)

The deviance information criterion (DIC) is a popular criterion for model choice^40^. It takes into account goodness-of-fit and a penalty term that is based on the complexity of the model via the estimated effective number of parameters. The DIC is defined as

$$\begin{aligned} DIC = D({\hat{x}}, \hat{\theta }) + 2pD \end{aligned}$$

where, D(.) is the deviance, ${\hat{x}}$ and $\hat{\theta }$ the posterieor expectations of the latent effects and hyperparameters, respectively, and pD is the effective number of parameters. The effective number of parameters pD can be computed as

$$pD = E[D(.)] - D({\hat{x}}, \hat{\theta })$$

The Watanabe-Akaike information criterion, also known as widely applicable Bayesian information criterion, is similar to the DIC but the effective number of parameters is computed in a different way. The final formula to calculate WAIC is.

$$\begin{aligned} WAIC = -2\sum _{i =1}^{n}log p_{post(y_{i})} + 2pD \end{aligned}$$

Where, $\sum _{i =1}^{n}log p_{post(y_{i})}$ is the sum of predictive density for each data point and pD is the effective number of parameters.

### Model diagnosis

#### Diagnosis for the accuracy of INLA approximation for the models

*The Kullback-Leibler divergence (kld):* This value describes the difference between the normal approximation and the simplified Laplace approximation. Small values indicate that the posterior distribution is well-approximated by a normal.

*Effective number of parameters(pD):* The posterior summary results from INLA also contain, effective number of parameters which is another measure of the accuracy of approximation. In particular, if the effective number of parameters is low compared to the sample size, then one expects the approximation to be good.

#### Goodness of fit test

A two types of “Goodness of fit” reported by INLA are:

*Conditional predictive ordinates (CPO)* Conditional predictive ordinates are a cross-validatory criterion for model assessment^41^. It is computed for each observation as

$$\begin{aligned} CPO_{i} = \pi (y_{i}/y_{-i}) \end{aligned}$$

Unusually small or large values of $CPO_{i}$ indicate a surprising observation.

*Predictive integral transform (PIT):* The predictive integral transform (PIT) measures the probability of a new value to be lower than the actual observed value for each observation^42^. It is computed as

$$\begin{aligned} PIT_{i} = \pi (y_{i}^{new} \le y_{i}/y_{-i}) \end{aligned}$$

An unusual large or small value indicates possible outliers.

Due to how $${\tilde{\pi }}(x_{i}/y_{-1}, \theta )$$ are computed there may be cases where this computation fails due to inaccurate tail behavior of $${\tilde{\pi }}(x_{i}/y_{i}, \theta _{j})$$. To monitor the reliability of the CPO and PIT values computed, failure variable computed for each i (or $$y_{i})$$ is defined as follows.

- If ${\tilde{\pi }}(x_{i}/y_{i}, \theta _{j})$ is monotone increasing or decreasing, then failure is set to 1 and then ${\tilde{\pi }}(x_{i}/y_{i}, \theta _{j})$ is set to the 0-function. In this case, ${\tilde{\pi }}(x_{i}/y_{i}, \theta _{j})$ is known to be just wrong.
- If ${\tilde{\pi }}(x_{i}/y_{i}, \theta _{j})$ is has a (local) maximum either at min$x_{i}$ or at max*x* *i*, then ${\tilde{\pi }}(x_{i}/y_{i}, \theta _{j})$ is set to zero in that part where ${\tilde{\pi }}(x_{i}/y_{i}, \theta _{j})$ is decreasing (starting from min($x_{i}$) or increasing (starting from max$x_{i}$. When the expected failure is 0 then the computed value of CPO and PIT seems to be reliable, and when the expected failure is 1 then the computed value of CPO and PIT is known to be completely unreliable.

### R-INLA

The statistical analysis was performed using R-INLA software package. R-INLA which is available at http://www.r-inla.org/ the R package through which the Bayesian inference with INLA methodology is implemented.

## Results

### Summary of descriptive statistics results

A total of 421 adult HIV/AIDS patients at ART clinic of Jimma Medical Center, Ethiopia were included in the analysis. During the follow-up period, 111(26.37%) of the study subjects had experienced the event(had been co-infected with TB.

The descriptive results of demographic and clinical characteristics of patients were presented in Table 1. The percentage of female patients who had been co-infected with TB was 53.15% which is larger compared to male patients. About 76.58% of the HIV patients with TB cases were urban residents. Among the patients, who had TB co-infection, about 36.94% were smokers and 57.67% were alcoholics. Patients with no education accounted for 14.41% of experiencing TB , patients with primary education accounted for 22.52%, patients with secondary education accounted for 39.63%, patients with Tertiary education accounted for 18.91%, and patients with education level of Diploma and above accounted for 4.50% of experiencing TB during follow up time. Among 111 HIV patients, who had TB co-infection, 16.22% of them are in WHO clinical I, 19.82% were in WHO clinical stage II, 27.02% were in WHO clinical stage III , and 36.94% were in WHO clinical stage IV. About 28.83%, 28.83%, and 42.34% co-infection of TB were occurred in working, ambulatory and bedridden HIV patients respectively.

**Table 1:** Descriptive results of the demographic and clinical characteristics of patients.

Covariates	Category	Patient Status		Total
		Censored	Event	
Sex	Male	128(71.1%)	52(28.9%)	180
	Female	182(75.5%)	59(24.5%)	241
Residence	Urban	159(65.2%)	85(34.8%)	244
	Rural	151(85.3%)	26(14.7%)	177
Smoking	No	256(78.5%)	70(21.5%)	326
	Yes	54(56.8%)	95(43.2)	95
Alcohol	No	204(81.3%)	47(18.7%)	251
	Yes	106(62.4%)	64(37.6%)	170
Education level	No formal education	42(72.4%)	16(27.6%)	58
	Primary education	71(74.0%)	25(26.0%)	96
	Secondary education	105(70.5%))	44(29.5%)	149
	Tertiary education	56(72.7%)	21(27.3%)	77
	Other	36(87.8%))	5(12.2%)	41
Family size	$\le$ 2	173(83.6%)	34(16.4%)	207
	3-4	116(69.5%)	51(31.5%)	167
	$\ge$ 5	21(44.7%)	26(55.3%)	47
Marital status	Single	43(63.23%)	25(36.77%)	65
	Married	152(76.8%)	46(23.2%)	198
	Widowed/Divorced	115(74.2%)	40(25.8%)	155
WHo disease stage	I	98(84.5%)	18(15.5%)	116
	II	80(78.4%)	22(21.6%)	102
	III	80(72.7%)	30(27.3%)	101
	IV	52(55.9%)	41(54.1%)	93
Functional status	Working	148(82.2%)	32(17.8%)	180
	Ambulatory	90(73.8%)	32(26.2%)	122
	Bedridden	72(60.5%)	47(39.5%)	119
CD4 count	$<200$	71(61.2%)	45(38.8%)	116
	200–349	71(64.5%)	39(35.5%)	110
	350–499	70(84.3%)	13(15.7%)	83
	$\ge 500$	98(87.5%)	14(12.5%)	112
BMI	Underweight	57(55.3%)	46(44.7%)	103
	Normal	190(82.3%)	41(17.7%)	231
	Overweight	63(72.4%)	24(17.6%)	87
Hemoglobin level	Anemic	18(45.0%)	22(55%)	40
	Moderate anemic	59(70.2%)	25(29.8%)	84
	Normal	233(78.5%)	64(21.5%)	297
	**Total**	**310(78.37%)**	**111(26.37%)**	**421(100%)**

Significant values are in [bold].

### Kaplan-Meier estimate of survival functions

**Figure 1:**
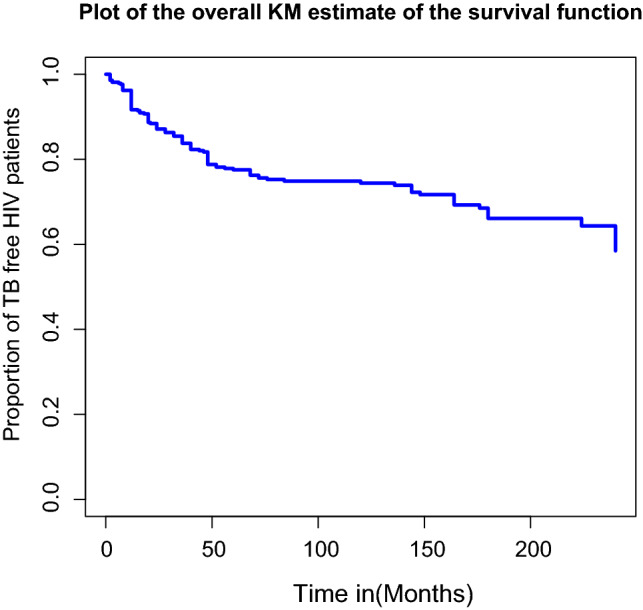
The overall Kaplan-Meier survival curve estimate of TB free co-infection survival time of HIV/AIDS patients.

**Figure 2:**
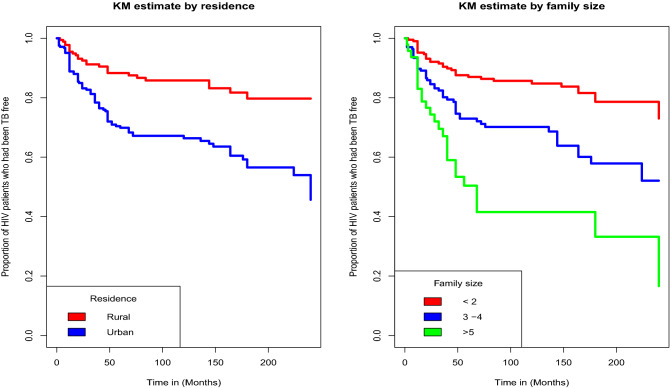
Kaplan-Meier survival curves for TB free co-infection survival time of patents by residence of patients and family size of patients.

**Figure 3:**
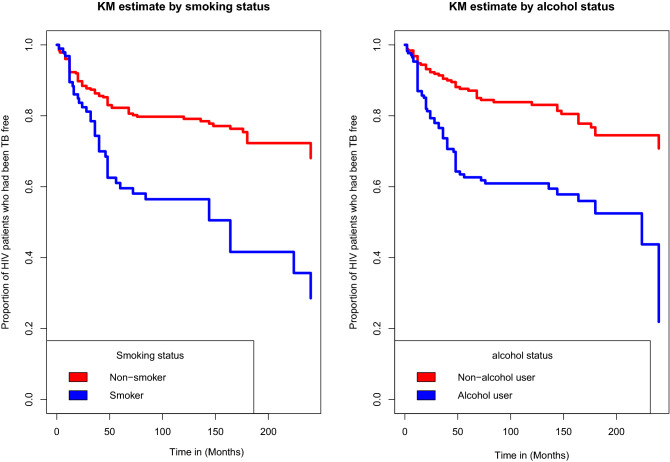
Kaplan-Meier survival curves for TB free co-infection free survival time of patients by smoking status and alcohol status of patients.

**Figure 4:**
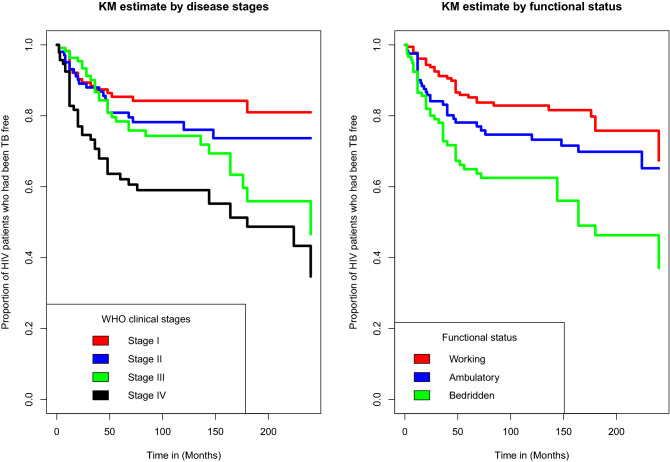
Kaplan-Meier survival curves for TB free co-infection free survival time of patients by disease stages and functional status of patients.

**Figure 5:**
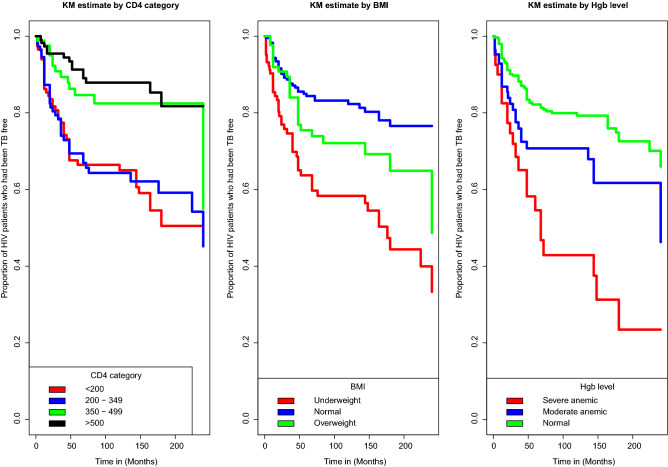
Kaplan-Meier survival curves for TB free co-infection survival time of patients by CD4 category, BMI and Hemoglobin level of patients.

From the plot of the overall Kaplan-Meier survival curve given in the Fig. 1 below, it can be seen that, a large number of TB co-infection recorded at the earlier time of the initiation of ART and there is a decreasing pattern of TB co-infection through the follow up period. In order to explore differences between TB co-infection free survival time between or among groups, separate KM survival function curves were constructed for categorical covariates and results are given in Figs. 2, 3, 4 and 5. In general, if the pattern of one survivor-ship function is above the other, it means the group defined by the upper curve had a better survival than the group defined by the lower curve.

### Comparison of Bayesian parametric survival models

Parametric AFT survival models(Exponential, Weibull, Log-logistic, Log-normal and Gamma modes) based on Bayesian paradigm considering all covariates were fitted for the data. In order to compare and select the best model among different parametric models, DIC, WAIC and Marginal likelihood of the models were used. The model with the smallest values of DIC and WAIC, and largest value marginal log-likelihood is selected as the best model. The five parametric survival models with their corresponding values of DIC, WAIC and Marginal LIkelihood values are displayed in Table 2. The Bayesian Log-logistic AFT model was found to be the best fitting model for our data set as it has the smallest values of DIC and WAIC, and has the largest values of marginal loglikelihood among the five models .

**Table 2:** Parametric survival models with their corresponding DIC, WAIC and Marginal log-likelihood.

Models	pD	DIC	WAIC	Marginal loglikelihood
Exponential	32.36	664.26	676.03	− 432.32
Weibull	29.97	659.73	667.72	− 427.32
Log-logistic	27.99	655.65	659.64	− 421.66
Log-normal	25.05	663.06	663.69	− 432.07
Gamma	27.68	664.19	670.29	− 430.14

## Assessment of factors associated with time to active TB co-infection in HIV/AIDS patients

### Results of Bayesian Log-logistic AFT model

Table 3 shows the results of the posterior summary results of Bayesian Log-logistic AFT model. The decision about the significance of the variables is based on the 95% Credible Interval for the posterior mean of the coefficients.

**Table 3:** Summary results of Bayesian Loglogistic AFT model.

Covariates	Categories	Mean	St. Dev.	Acceleration factor($\hat{\gamma }$)	95% Credible Interval
Age	Intercept	6.460	0.937		[4.658, 8.333]
	Continuous	0.007	0.014	1.0070	[− 0.020, 0.035]
Sex	Female	− 0.189	0.285	0.8278	[− 0.748, 0.373]
	Male(Ref.)				
Residence	Urban	− 1.258	0.297	0.2842	[− 1.851, − 0.686]*
	Rural(Ref.)				
Smoking	Yes	− 0.667	0.308	0.5137	[− 1.268,− 0.061]*
	No(Ref.)				
Alcoholics	Yes	− 0.879	0.295	0.4151	[− 1.459, − 0.300]*
	No(Ref.)				
Education levels	No education(Ref.)				
	Primary education	0.033	0.427	1.0335	[− 0.811, 0.866]
	Secondary education	0.102	0.398	1.1074	[− 0.688, 0.874]
	Tertiary education	0.610	0.469	1.8404	[ − 0.308, 1.531]
	Diploma & above	1.055	0.670	2.8720	[− 0.213, 2.417]
WHO clinical stages	Stage I(Ref.)				
	Stage II	− 0.472	0.408	0.6238	[− 1.278, 0.323]
	Stage III	− 0.849	0.380	0.4278	[− 1.605, − 0.113]*
	Stage IV	− 1.106	0.379	0.3308	[− 1.860, − 0.371]*
Functional status	Working(Ref.)				
	Ambulatory	− 0.502	0.345	0.6053	[− 1.181, 0.173 ]
	Bedridden	− 0.672	0.315	0.5107	[− 1.291, − 0.056]*
Family size	$\le 2$(Ref.)				
	3–4	− 0.933	0.308	0.3933	[− 1.543, − 0.332]*
	$\ge 5$	− 1.131	0.384	0.3227	[− 1.885, − 0.379]*
CD4 count	$< 200$	− 1.534	0.410	0.2156	[− 2.356, − 0.746]*
	200–349	− 0.980	0.421	0.3753	[− 1.820, − 0.168]*
	350–499	− 0.220	0.479	0.8025	[− 1.157, 0.723]
	$$\ge 500$$(Ref.)				
BMI	Underweight	− 0.950	0.300	0.3867	[− 1.541, − 0.363]*
	Normal(Ref.)				
	Overweight	− 0.427	0.350	0.6525	[− 1.110, 0.264]
Marital Status	Married(Ref.)				
	Single	− 0.543	0.389	0.5810	[− 1.300, 0.224]
	Widowed/Divorced	− 0.120	0.307	0.8869	[− 0.722, 0.482]
Hemoglobin	Severe anemic	− 1.192	0.392	0.3036	[− 1.960 , − 0.421 ]*
	Moderate anemic	0.080	0.354	1.0832	[− 0.604, 0.785]
	Normal(Ref.)				

Based on Bayesian Log-logistic AFT model results, it appeared that residence, smoking status, alcohol consumption status, WHO clinical stages, functional status, family size, CD4 count, BMI and hemoglobin level of the patients were significant risk factors associated with time to TB co-infection of HIV/AIDS patients at Jimma University Medical Center. The interpretation of the estimated posterior mean of parameters of the model was done using estimated acceleration factor($\hat{\gamma } = exp(\beta _{j}$). In order to decide the significance of the covariates in the model, the 95% credible interval was used. The factors whose credible intervals for posterior mean of parameters contained 0, or whose credible intervals for acceleration factor contained 1, implied that these factors were not significant. The results of the final model can be written as:

$$\begin{aligned}&log(T_{i}) = 6.460 - 1.258I_{\text {(Residence} = 2)} - 0.667I_{\text {(Smoking} = 2)} - 0.879I_{\text {(Alcoholics} = 2)} - 0.472I_{\text {(Clinstages} = 3)}- 0.849I_{\text {(Clinstages} = 4)} \\&\quad \quad-0.672I_{\text {(Funstat} = 3)}- 0.980I_{\text {(Famsize} = 3 - 4)}- 1.131I_{\text {(Famsize}\ge 5)}- 1.534I_{\text {(CD4} = 3)} - 0.980 I_{\text {(CD4} = 4)} - 0.950I_{\text {(BMI} =2)} \\&\quad \quad-1.192I_{\text {(Hgb} =2)} \end{aligned}$$

where, T represents time to TB co-infection for each subject. I is an indicator variable for categories of variables where $I_{(. = 1)}$ is considered as a reference category.

In Log logistic AFT model, the positive estimated posterior $\beta$ coefficients indicate a longer TB co-infection free survival time, where as the negative estimated posterior $\beta$ coefficients indicate shorter TB co-infection free survival time for the patients.

The estimated acceleration factor for patients who reside in urban was estimated to be $\hat{\gamma }$ = exp(− 1.258) = 0.2842 with 95% CI of $\left[ 0.1571, 0.5035\right]$. This means that, keeping all other factors constant the expected TB co-infection free survival time of patients who reside in urban area decreases by a factor 0.2842 as compared to patients residing in rural area.

The estimated acceleration factor of smoker patients was 0.51237(95% CI: 0.2814, 0.9361]. This indicates that patients who smoke had shorter TB co-infection free survival time, compared to patients who were not smokers. This is the same with patients who drink alcohol with an estimated acceleration factor of 0.4151(95% CI: 0.2324, 0.7408). Accordingly, it can be said that patients who drink alcohol had shorter time to develop(to be co-infected) with TB.

It was found that advanced clinical stages(stage III($\hat{\gamma }$ = 0.4278[95% CI: 0.2009, 0.8932]) and stage IV($\hat{\gamma }$ = 0.3308[0.1556, 0.6900]) led to a decrease in TB co-infection free survival time. Patient with bedridden functional status($\hat{\gamma }$ = 0.5107(95% CI of [0.2750, 0.9455]) had also a shorter TB co-infection free survival time, when compared to patients with working functional status at baseline. CD4 counts($\le 200$cells/mm^3^ with an acceleration factor of 0.2156[95% CI:0.0948, 0.4742] and $200 - 349$cells/mm^3^ with an acceleration factor of 0.3753[95% CI:0.1620, 0.8453] also found to shorten TB co-infection free survival time, as compared to patients with CD4 counts $\ge 500$cells/mm^3^.

When we look at relationship between family size and time to TB co-infection, those patients with family size of 3–4($\hat{\gamma }$= 0.3933[95% CI:[0.2137, 0.7174] and family size of $\ge 5$($\hat{\gamma } = 0.3227[95\% CI: 0.1518, 0.6845]$ had shorter TB co-infection free survival time than those with family size of $\le 2$.

The estimated acceleration factor for underweight patients was 0.3667[95% CI: 0.2141, 0.6955], and the estimated acceleration factor of severe anemic patients was estimated to be 0.3036[95% CI: 0.1408, 0.6564]. Subjects who were underweight and severely anemic had shorter TB co-infection free survival time.

It can also be seen that patients with low body mass index and patients with severe anemic status was found to be the significant risk factors for TB co-infection in HIV patients. The estimated acceleration factor for underweight patients was 0.3667 with 95% CI of [0.2141, 0.6955], and the estimated acceleration factor of severe anemic patients was estimated to be 0.3036 with CI of [0.1408, 0.6564]. Thus, keeping all other factors constant, the TB co-infection free survival time of underweight HIV patients decreases by a factor of 0.3667 as compared to patients with normal weight,and, the TB co-infection free survival time of severe anemic HIV patients decreases by a factor of 0.3036 as compared to patients with normal anemic status.

### Model diagnosis results

*The Kullback-Leibler divergence (kld):* For the model above the values of kld for each coefficient was zero which means the marginal posterior densities of regression coefficients were well approximated by the Normal distribution.

*Effective number of parameters(pD):* In this study, the ratio of sample size (421) and effective number of parameters (28.93) was found to be 14.55, suggesting a reasonably good approximation. The ratio can be interpreted as the number of equivalent replicates corresponding to the number of observations for each expected number of effective parameters.

## Discussions

Among the study participants who fulfilled the inclusion criteria, about 26.37% of subjects had been co-infected with Tuberculosis. The proportion of of TB co-infection in this study cohort is smaller compared to other study settings in different parts of Ethiopia in which it was found to be 62.3% and 40.1% in a retrospective study conducted in seven ART clinics located at Addis Ababa and in North-east Ethiopia with respectively.The possible reason for finding lower proportion of TB co-infection in this study setting might be due to the fact that there are disparities in environmental factors. The discrepancy may also be attributed to TB/HIV co-infection management. The proportion of TB co-infection observed in this study is consistent with the findings from Noth-west Ethiopia(26.4%)^18^, Amhara region(27.7%)^10^.

The findings of this study showed that patients’ residence place were significantly associated with TB co-infection free survival time of HIV/AIDS patients. Patients who reside in urban areas are more likely to be infected with TB as compared to patients residing in rural areas. TB co-infection free survival time for patients who reside in urban areas were found to be shorter than those who reside in rural areas. This result is consistent with the report of the retrospective study conducted by Beshir et al. at Adama Referral Hospital and Medical College, Oromia, Ethiopia^43^. They reported residence place as one of the significant risk factors of TB co-infection in HIV/AIDS patients. This may be due to the fact that there is overcrowding of people in urban areas than in the rural areas. However, the finding of this study is inconsistent with those of other studies undertaken in Ethiopia^13,18,44^.

It was found that Smoker and alcohol user patients had shorter TB co-infection free survival time than those who are not smoker and non-alcohol users. The result of the study agrees with the result reported by Anye et al. based on four year retrospective data of 1077 HIV patients in the Bameda regional hospital of Cameroon^45^. Our result also agrees with report of studies undertaken in Ethiopia^18,44,46^. Their results suggested that being smoker is significantly associated with TB co-infection free survival time in HIV/AIDS patients.This similarity might be due to the fact that the unhealthy life style, like smoking, alcohol consummations expose the patients to several opportunistic infections by facilitating the decline of patients’ immunity and shortening their opportunistic infection free survival time.

The result of our study suggested that baseline clinical stages were one of the clinical factors associated with TB co-infection free survival time of HIV/AIDS patients.Patients with advanced WHO clinical stages(stage III and stage IV) had shorter TB co-infection survival time than those with WHO clinical stage I. According to the findings of the study conducted by Patients with WHO clinical stages III and IV are more likely to be co-infected with TB than those with clinical stage I. This finding supports the findings of the study undertaken by Kebdeet al.^17^ being in advanced clinical stages is associated with higher risk of developing TB compared WHO clinical stages I and II^17^. Our finding also coincides with the study conducted in Amhara region of Ethiopia by Aweke et al.^10^. This might be due to the fact that once patients get into late stages, the immunity of an individual declines, making them infected with TB.

Similarly, in this study, patients’ functional status at baseline was found to be the predictor of TB co-infection free survival time of HIV/AIDS patients. This result is consistent with the report of the study conducted by Aemro et al. at Debra Markos referral hospital, Northwest Ethiopia^47^. Accorging to our study, patient with bedridden functional status at baseline had shorter TB co-infection free survival time compared to patients with working functional status at baseline. This might be due to the fact that lack of physical activity exposes them to many opportunistic diseases, including TB.

The findings of this study and the results of other studies conducted in Ethiopia and other countries indicated that, HIV patients with a lower CD4 counts at a baseline are at a higher risk of co-infection with TB^10,18,45^. HIV patients with CD4 counts($\le 200$ and 200–349 cell/mm^3^ had shorter TB free co-infection survival time. This might be attributed to the fact that CD4 count is an indicator of an individual immune function in HIV patients.

This study had revealed that HIV/AIDS patients who were underweight were at a risk of shorter TB co-infection free survival time. This result was consistent with the findings of study done by Ahmed et al. and Alemu in Ethiopia^18,44^. The possible reason for this might be due to the fact that low BMI is an indicator of malnutrition which leads HIV patients to increased catabolism, loss of appetite which further increase the risk of infection with TB.

The findings of this study also showed that HIV patients with severe anemic status were responsible for decreasing TB co-infection free survival time of HIV patients compared to patients with normal anemic status.This finding is consistent with the result of the study conducted by Alemu et al., Brenan et al., Kebede et al. in Adama Hospital^43^, South Africa^48^, Benishangul Gumuz region, Northwest of Ethiopia^17^. This might be due to the fact that anemia leads to complications of both TB and HIV infection.

This study employed Bayesian parametric AFT survival models(Exponential, Weibull, Log-logistic, Log-normal and Gamma) to model time to TB co-infection in HIV/AIDS patients using INLA methodology. The main advantage of AFT survival models is that AFT model directly models the time to event data rather than hazard ratios, which makes the interpretation of the results clinically relevant. The model selection result of this study indicated that the log-logistic model is the best fitting model for the data set. Log-logistic model is more convenient survival model when dealing with censored data due to the fact that it has a more manageable shape^37^.

## Conclusion

Bayesian survival analysis approach with INLA methodology was applied to fit the parametric survival models to our data set. Among the parametric AFT survival models, Bayesian Log-logistic AFT model was found to be the best fitting model for our data set.

Place of residence, smoking, drinking alcohol, larger family size, advanced clinical stages(Stage III and Stage IV), bedridden functional status, CD4 count($\le$ 200 cells/$mm^{3}$ and 200–349 cells/$mm^{3}$, low BMI and low hemogilobin are the factors that lead to shorter TB coinfection survival time in HIV/AIDS patients.

### Recommendation

The findings of the study suggested us to forward the recommendations to modify patients’ life style, early screening and treatment of opportunistic diseases like anemia , as well as effective treatment and management of TB/HIV co-infection are important to prevent TB/HIV co-infection.

### Limitation of the study

The limitation of this study is that the results in this study was based Jimma Medical Center only. We have not considered other hospitals in Jimma town. The other limitations of this study is that some important factors like ART adherence status, ART treatment regimen, diabetes mellitus, viral load and hypertension that could potentially affect TB and HIV co-infection had not been considered. The study also does not take into account the types of tuberculosis. Also, ur results based on data of ART clinic of Jimma Medical Center need to be substantiated by similar survival studies from other parts of Ethiopia to give a comprehensive picture of TB and HIV co-infection in the country. Regardless of these limitations, our findings have policy implications and can be used as reference in future studies.

## Data Availability

The data sets used during the current study available from the corresponding author on reasonable request.

## Abbreviations

AIC: Akaike Information criteria
AFT: Accelerated Failure Time Model
ART: Anti-Retroviral Therapy
AIDS: Human Immuno Deficiency Syndrome
BIC: Bayesian Information Criteria
BMI: Body Mass Index
CPT: Cotrimoxazole Preventive Therapy
Cox-PH: Cox Proportional Hazard model
DIC: Deviance Information Criteria
FMOH: Federal Ministry of Health
HIV: Human Immunodeficiency Virus
INLA: Integrated Nested Laplace Approximation
IPT: Ionized Preventive Therapy
KLD: Kullback Leibler Deviance
MCMC: Markov Chain Monte Carlo
MLE: Maximum Likelihood Estimate
OI: Opportunistic Infection
SE: Standard Error
PH: Proportional Hazard
PLHIV: People Living with HIV
SSA: Sub-Saharan Africa
TB: Tuberculosis
WAIC: Widely Applicable Information Criteria
WHO: World Health Organization
